# Food Folate Quality: Linking Dietary Sources, Bioavailability, and Human Health

**DOI:** 10.3390/foods15213797

**Published:** 2026-10-23

**Authors:** Chih-Chen Wang, Bi-Fong Lin, Chia-Chien Hsieh

**Affiliations:** Department of Biochemical Science & Technology, National Taiwan University, No. 1, Sec. 4, Roosevelt Rd., Taipei 10617, Taiwan; f13b22010@ntu.edu.tw

**Keywords:** natural folate, folic acid, food sources, bioavailability, folate status, health effects

## Abstract

Food folate concentrations do not necessarily predict the amount available for human metabolism. This narrative review summarizes how dietary sources, analytical methods, and digestive processes influence interpretations of food folate quality. Literature was identified through PubMed using searches covering 2000–2026, selecting studies relevant to food sources, measurement, bioavailability, and human health. We propose a framework linking food folate, analytical reliability, bioaccessibility, absorption, and biological efficacy. Major sources include leafy vegetables, legumes, fruits, grains, and liver, but folate content varies with food type, origin, and processing. Natural food folates require matrix release and polyglutamate hydrolysis before absorption, limiting comparisons based on concentration alone. Adequate folate intake is closely linked to reduced risks of folate-deficiency anemia and fetal neural-tube defects, and some studies have investigated its role in regulating cardiovascular, cognitive, metabolic, immune, and cancer-related effects. Adverse effects of high folic acid exposure depend on dose, vitamin B_12_ status, and study design; the role of circulating unmetabolized folic acid remains uncertain. Overall, a diverse diet rich in natural folate supports nutritional adequacy, while targeted fortification and supplementation need to avoid excessive exposure. Evaluating food folate quality requires both accurate measurements and evidence of bioavailability.

## 1. Introduction

Folate is an essential water-soluble vitamin, also known as vitamin B_9_, that is required for nucleotide synthesis, amino acid metabolism, methyl-group transfer, and normal cell proliferation. As a central component of one-carbon metabolism, folate supports de novo purine and thymidylate synthesis, homocysteine remethylation, methionine regeneration, and production of S-adenosylmethionine, the principal methyl donor involved in cellular methylation reactions [1,2]. Folate requirements are particularly critical during periods of rapid growth and cell division, including pregnancy, infancy, childhood, and adolescence. Inadequate folate status can impair DNA synthesis and erythropoiesis, resulting in megaloblastic anemia [3]. Moreover, insufficient folate intake during early pregnancy is associated with an increased risk of fetal neural-tube defects (NTDs) [4].

Dietary folates occur naturally in a wide range of foods, particularly dark-green leafy vegetables, legumes, whole grains, and liver. Several fruits, including citrus fruits, tropical fruits, strawberries, and avocados, are also important sources of folate, although their contribution is often overlooked. In contrast, synthetic folic acid is an oxidized and relatively stable monoglutamate form that is widely used in food fortification and supplements. Most naturally occurring food folates are polyglutamates and must be released from the food and hydrolyzed to the monoglutamate before absorption [5,6]. These structural and chemical differences may affect folate stability, release during digestion, intestinal absorption, and metabolic utilization. Consequently, naturally food folates and synthetic folic acid are not necessarily nutritionally equivalent [7,8].

Food composition databases commonly list folate concentrations in foods; however, these values do not necessarily reflect the amount of folate that is ultimately available for absorption and physiological utilization. Reported folate concentration can vary substantially according to food species and cultivar, stage of maturity, geographical origin, storage conditions, extraction efficiency, enzymatic deconjugation, and analytical methodology. Among the analytical methodology used for folate determination, microbiological assays assess the growth response of folate-dependent microorganisms and have traditionally been used to estimate total folate content. In contrast, high-performance liquid chromatography (HPLC) and liquid chromatography–tandem mass spectrometry (LC-MS) can separate and quantify individual folate vitamers, providing more detailed information on folate composition [9,10]. Consequently, folate values obtained using different analytical methods may not be directly comparable. Moreover, foods with similar total folate concentrations may differ substantially in folate vitamer composition, polyglutamate distribution, localization of food, and digestibility. The release, absorption, and utilization of folate are influenced not only by food structure and but also by individual factors, including gastrointestinal health, alcohol consumption, and genetic variation [8]. Therefore, evaluating food folate quality based solely on food composition databases may be misleading in terms of the nutritional value of dietary folates.

Bioavailability represents another important challenge in the evaluation of dietary folates. Naturally occurring food folates are generally less bioavailable than synthetic folic acid because they are predominantly present as polyglutamates and must be undergo release, deconjugation, and subsequent metabolic processing before utilization. This difference in bioavailability is reflected in the concept of dietary folate equivalents (DFEs) [7,11]. Nevertheless, the bioavailability of food folates varies considerably among food types and is influenced by food matrix characteristics, preparation and processing, and individual physiological factors [5,12]. At the same time, increasing exposure to folic acid through fortified foods and dietary supplements has raised questions regarding the potential consequences of excessive intake. These concerns include the potential for folic acid to mask hematological manifestations of vitamin B_12_ deficiency and possible associations with adverse health outcomes, including cancer, although the evidence remains complex and context-dependent. In addition, the physiological significance of unmetabolized folic acid detected in the circulation remains incompletely understood [13,14]. Taken together, these considerations highlight the importance of distinguishing naturally occurring food folates from synthetic folic acid when evaluating dietary folate exposure and human health.

Global nutritional surveillance indicates that inadequate folate intake remains common, particularly among women of reproductive age, and contributes to preventable cases of megaloblastic anemia and neural-tube defects [3,4]. These findings support the need for reliable food composition databases and effective strategies to improve folate intake. In addition, folate status and folic acid supplementation have also been investigated in relation to cardiovascular disease, cognitive decline, metabolic dysfunction, inflammation, and cancer. However, evidence for these outcomes remains less consistent than that supporting the established benefits of folate in preventing folate-deficiency anemia and NTDs [6,15,16].

A central challenge in folate nutrition is determining how accurately measured food concentrations translate into biological availability and health benefits. Differences in food sources, analytical methods, vitamer forms, exposure forms, and outcome measures complicate comparisons across studies. This narrative review addresses these limitations by proposing a framework for evaluating food folate quality that links folate concentration and analytical reliability with digestive release, bioaccessibility, absorption, and biological efficacy. It specifically connects food composition data with the evidence needed to interpret nutritional value beyond total folate content.

To integrate this review, relevant studies from 2000 to 2026 were identified through the PubMed database. We identified and selected keywords relevant to folate, food sources, analytical methods, bioavailability, bioaccessibility, and human health. Eligibility criteria included SCI-indexed reviews and original papers. In the human health section, selected articles focused more on observational studies, human intervention evidence, and their systematic reviews, which are closer to application.

This review examines dietary folates across the food-to-health pathway. It first describes the chemistry and biological functions of major folate forms. It then summarizes dietary sources of folate, their distribution, and methods of measurement in foods. Next, it evaluates the effects of food-matrix characteristics, polyglutamate deconjugation, and host factors on folate bioaccessibility and bioavailability. Finally, the review examines the relationships among dietary folate intake, nutritional status, and human health. Overall, this review provides a basis for evaluating folate-rich foods and informing dietary guidance while identifying gaps between chemical measurement and demonstrated physiological benefit.

## 2. Chemistry and Biological Functions of Dietary Folates

### 2.1. Folate Nomenclature and Chemical Characteristics

Folate, also known as vitamin B_9_, refers to a group of structurally related compounds comprising three principal components: a pteridine ring containing nitrogen, para-aminobenzoic acid, and one or more glutamate residues. Their chemical diversity arises from differences in oxidation state of pteridine ring, the position of one-carbon substituents—primarily at the N5 and N10 positions—and the number of glutamate residues [1,17]. These structural characteristics influence the biochemical functions, stability, cellular retention, and nutritional availability of individual folate forms.

The term folate should be distinguished from folic acid. Folic acid, or pteroylmonoglutamic acid, is a fully oxidized, relatively stable synthetic form used primarily in fortified foods and dietary supplements. Before entering one-carbon metabolism, folic acid must be reduced by dihydrofolate reductase to dihydrofolate and subsequently to tetrahydrofolate (THF). In contrast, folates naturally present in foods occur predominantly as reduced THF derivatives. Following intestinal absorption and intracellular conversion, these compounds can enter folate-dependent metabolic pathways [5,18]. Thus, folic acid differs from food folates in terms of its origin, chemical stability, metabolic activation, and potential systemic exposure.

Folic acid and circulating folates occur predominantly as monoglutamates, whereas folates in foods and cells commonly occur as polyglutamates. Dietary polyglutamates, however, must first be hydrolyzed to absorbable monoglutamates by intestinal glutamate carboxypeptidase II before absorption [19]. Following cellular uptake, folates are converted back to polyglutamate forms by folylpolyglutamate synthetase. Polyglutamation promotes intracellular folate retention and facilitates the compartmentalization of folates within the cytosol, mitochondria, and nucleus [20].

The reduced structure of natural folates renders them chemically less stable than folic acid. THF and several of its one-carbon derivatives are susceptible to oxidation, cleavage, and interconversion, particularly under unfavorable conditions involving pH, temperature, oxygen exposure, and light. This instability has important implications for folate determination in foods, as sample handling, storage, and processing can alter both the concentrations measured and the distribution of individual folate forms [5,21].

### 2.2. Major Folate Vitamers in Foods

Naturally occurring food folates comprise several chemically reduced forms, including THF, 5-methyltetrahydrofolate (5-MTHF), 5-formyltetrahydrofolate (5-Formyl-THF), 5,10-methylenetetrahydrofolate, and 10-formyltetrahydrofolate, with their relative proportions varying by food type [5,22]. THF is a central metabolically active folate coenzyme but is relatively unstable and is generally present in foods at low concentrations. Within cells, THF derivatives function as carriers of one-carbon units and participate in a range of folate-dependent metabolic reactions. Among food folates, 5-MTHF is generally the predominant form and is also the major circulating folate vitamer. It serves as the methyl donor for the remethylation of homocysteine to methionine, a reaction that requires vitamin B_12_ as a cofactor. Under conditions of vitamin B_12_ deficiency, folate can become metabolically trapped as 5-MTHF, thereby limiting its availability for other folate-dependent reactions [23,24]. 5-Formyl-THF, also known as folinic acid, is a relatively stable folate derivative that can function as a storage form and contribute to the regulation of intracellular folate availability; its importance may be particularly relevant during pregnancy, when folate requirements increase [25]. These folate vitamers are metabolically interconnected, and the composition of the body’s folate pool reflects both dietary intake and subsequent absorption, metabolism, and interconversion.

Folate vitamer profiles also differ between naturally occurring and fortified foods. Natural foods predominantly contain reduced folate forms, whereas fortified foods generally contain added folic acid and, in some cases, 5-MTHF salts. Because these folate forms differ in chemical stability, metabolic requirements, and absorption characteristics, measurements reported as “total folate” may obscure nutritionally relevant differences in folate composition [18,22].

### 2.3. Biological Functions of Folate

Folate supports a range of essential biological functions through one-carbon metabolism, which operates across interconnected cytosolic, mitochondrial, and nuclear pathways and contributes to nucleotide synthesis, amino acid metabolism, methylation reactions, and cellular redox homeostasis [2,26]. These processes are particularly important in rapidly proliferating cells. Within mitochondria, one-carbon metabolism generates formate from serine and glycine. Formate can then be transported to the cytosol, where it supplies one-carbon units for nucleotide biosynthesis and other folate-dependent reactions. The compartmentalization of folate metabolism enables the coordinated utilization of one-carbon units across distinct biosynthetic pathways and allows metabolic flux to respond to cellular growth and stress conditions [2,26].

A major function of folate is to support DNA and RNA synthesis through de novo purine biosynthesis and thymidylate synthesis. 10-Formyltetrahydrofolate donates one-carbon units at two steps in purine ring formation, whereas 5,10-methylenetetrahydrofolate provides a methylene group for the thymidylate synthase-catalyzed conversion of deoxyuridine monophosphate to deoxythymidine monophosphate. During this reaction, 5,10-methylenetetrahydrofolate is oxidized to dihydrofolate, which must subsequently be reduced to tetrahydrofolate by dihydrofolate reductase. Folate deficiency can therefore impair thymidylate synthesis, leading to increased uracil misincorporation into DNA, DNA strand breaks, and genomic instability [1,2]. These functions help explain the particular sensitivity of rapidly dividing tissues, including bone marrow and embryonic tissues, to inadequate folate availability.

Folate also plays a central role in methionine and homocysteine metabolism. In the methionine synthase reaction, 5-MTHF donates a methyl group to homocysteine, thereby regenerating methionine. Methionine is subsequently converted to S-adenosylmethionine, a major cellular methyl donor involved in the methylation of nucleic acids, proteins, lipids, and other biomolecules. Following methyl-group transfer, S-adenosylmethionine is converted through the methionine cycle, ultimately regenerating homocysteine. Thus, folate availability influences homocysteine metabolism and contributes to the maintenance of cellular methylation capacity [2,23]. Folate and methionine metabolism are closely integrated with other one-carbon nutrients, including vitamins B_2_, B_6_, and B_12_, as well as choline. Vitamin B_2_ is required for methylenetetrahydrofolate reductase, the enzyme responsible for the formation of 5-MTHF. Vitamin B_6_ serves as a cofactor for enzymes involved in serine–glycine interconversion and the transfer of one-carbon units to folate. Vitamin B_12_ is an essential cofactor for methionine synthase and is therefore required for the utilization of 5-MTHF in homocysteine remethylation. Choline, through its oxidation product betaine, provides an alternative methyl donor for the remethylation of homocysteine, particularly in the liver and kidneys [1].

Given the diverse biological functions of folate, both adequate intake and the selection of dietary sources are important for maintaining folate status. The nutritional significance of foods depends not only on their total folate content but also on the specific folate vitamers present and their susceptibility to processing, digestion, absorption, and metabolism. The following sections therefore examine the mechanistic basis underlying differences in food folate composition and bioavailability, and potential health effects are discussed.

## 3. Dietary Source Distribution and Measurement of Food Folates

### 3.1. Natural Dietary Sources and Folate Distribution

Natural folates are widely distributed across foods, although their concentrations and vitamer profiles vary substantially among food groups. Food folates include THF, 5-MTHF, and formylated derivatives, often bearing multiple glutamate residues. Their proportions and chemical stability vary among foods. Liver is among the richest dietary sources of folate, followed by dark-green leafy vegetables, while legumes are also widely recognized as important sources of naturally occurring folate. Whole grains, nuts, seeds, eggs, dairy products, and other vegetables generally contain lower concentrations, but their frequent consumption can nevertheless make meaningful contributions to overall folate intake [5,6,27]. Accordingly, Table 1 summarizes selected vegetable sources, reported folate concentration ranges, and the corresponding research findings.

Folate concentrations within the same food variety can also vary considerably among geographical regions. Such variation may reflect differences in genotype, environmental conditions, soil characteristics, agricultural practices, cultivar, maturity, and post-harvest handling. Consequently, dietary folate intake is determined not only by the folate concentration of individual foods but also by serving size, consumption frequency, overall dietary patterns, and the availability and consumption of fortified foods.

Green vegetables, including spinach, asparagus, broccoli, and lettuce, can contain more than 100 µg folate/100 g and may therefore be considered important dietary sources of folate. Other vegetables, including light-colored varieties, also provide appreciable amounts of folate and can contribute to overall folate intake, particularly when consumed regularly as part of a diverse, plant-rich diet. Total folate concentrations and vitamer distributions vary among vegetables, with 5-MTHF frequently representing a major proportion of the total folate content [34,38].

Mushrooms are also of interest as potential dietary sources of folate. In addition to providing folate, mushrooms contain dietary fiber and structurally diverse polysaccharides that have been investigated for their potential gastrointestinal and immunomodulatory effects. Although mushrooms belong to the fungal kingdom and are therefore distinct from plant-based vegetables, they are commonly consumed as part of the human diet. Their folate concentrations vary among species and varieties, as shown in Table 2. Collectively, these data indicate that commonly consumed mushrooms may contribute to dietary folate intake and represent an additional food source of folate.

Fruits generally contain lower concentrations of folate than leafy vegetables and legumes, although certain fruits, including oranges, avocados, papayas, strawberries, and several tropical fruits, can provide appreciable amounts [38,40], as summarized in Table 3. To further characterize fruits as potential dietary sources of folate, we analyzed the folate contents of 25 commonly consumed fruits in Taiwan using a microbiological assay in our laboratory (Table 4). This investigation was motivated by changes in dietary patterns in Taiwan, including increasing fruit consumption alongside a decline in vegetable intake.

Overall, the analyzed fruits generally contained moderate amounts of folate, although several, including durian, atemoya, strawberry, cherry tomato, Kyoho grape, papaya, golden kiwifruit, and orange, exhibited relatively high folate concentrations that were comparable to those reported for some leafy vegetables. In addition to their folate content, fruits can be consumed with minimal preparation, which may facilitate their regular inclusion in the diet. Thus, even fruits with moderate folate concentrations may contribute meaningfully to habitual folate intake when consumed frequently and in sufficient amounts. However, because fruits also contribute dietary energy, excessive consumption may increase total energy intake. To provide a practical context for balancing folate intake with energy consumption, Table 4 also presents the folate content in relation to a standard serving of fruit, with one serving providing approximately 60 kcal (kilocalories) on average. This information may help evaluate fruits not only according to their folate concentration but also in terms of their contribution to overall dietary energy intake [45].

Legumes such as chickpeas, lentils, beans, soybeans, and peas also contain appreciable folate concentrations and may contribute moderately to a plant-based diet, as shown in Table 5. In addition, nuts and seeds provide moderate amounts of folate, as shown in Table 6, although their contribution to overall folate intake depends strongly on portion size and consumption frequency. Cereal grains, in contrast, are not intrinsically rich sources of folate, which is naturally concentrated in the bran and germ. Refining and milling processes remove substantial portions of these fractions and consequently reduce the folate content of the resulting products [46,47]. Nevertheless, cereals and cereal-based products can make important contributions to dietary folate intake because they are consumed frequently and, in many populations, in relatively large quantities. In countries with mandatory or voluntary folic acid fortification programs, fortified or enriched flour, bread, pasta, rice, and breakfast cereals may represent major sources of dietary folate equivalents [47]. When assessing the contribution of cereal products to folate intake, it is important to distinguish naturally occurring reduced folates from folic acid added during food fortification.

Among animal-derived foods, liver is an exceptionally rich source of naturally occurring folate, reflecting its central role in folate storage and metabolism, as shown in Table 7. However, liver is not routinely consumed in most dietary patterns. Eggs, dairy products, meat, fish, and seafood generally contain lower concentrations of folate but can nevertheless contribute to overall folate intake in omnivorous diets [14,51]. In milk, 5-MTHF is a major folate vitamer and is associated with folate-binding proteins that may influence folate stability and bioavailability [50]. Animal- and plant-derived foods also differ in their relative proportions of THF, methylated folates, and formylated derivatives, highlighting the importance of considering folate vitamer composition and distribution within the food matrix in addition to total folate content [52,53].

### 3.2. Variation in Food Folate Content

Folate levels vary considerably within the same food type. In plants, folate concentration is influenced by cultivar, maturity stage, plant tissue, environmental conditions, and agricultural practices. Folate biosynthesis and accumulation may respond to light exposure, temperature, nutrient availability, and physiological stress. Moreover, leaves, stems, roots, seeds, and fruits can differ in both total folate and vitamer composition [38,58]. In animal-derived foods, folate content may be affected by species, tissue, diet, physiological stage, and production system. These sources of variation limit the reliability of using a single composition value to represent samples.

Sample definition also affects reported folate concentrations. Values may be expressed on a fresh-weight, dry-weight, edible-portion, or serving basis, resulting in apparently different concentrations even when the analyzed materials are similar. In addition, composite samples used in national food surveys may provide estimates of the average food supply, whereas analysis of a single sample may be more comparable [28,29,44]. Variation among food-composition databases reflects both biological differences but also inconsistencies in data generation. Additionally, reported variation reflects differences in sample handling and analytical methods. Storage and cooking may reduce folate through degradation or leaching, with retention depending on the food and processing conditions [43,59]. Folate values are derived from direct chemical analysis, data from foreign databases, recipe calculations, or estimates based on similar foods. Some older databases were generated using methods that incompletely extracted polyglutamatyl folates or did not distinguish individual vitamers. Updating national databases with validated analytical data can substantially alter estimates of folate contents, as demonstrated by revisions to the Dutch Food Composition Database [52,60].

### 3.3. Sample Preparation and Extraction of Food Folates

Accurate measurement of food folate remains technically challenging because foods contain multiple labile vitamers, often as polyglutamates associated with complex food matrices. Folate losses may occur during sample collection, homogenization, storage, extraction, deconjugation, purification, and instrumental analysis, thereby affecting the final results. Exposure to light, oxygen, heat, or inappropriate pH may promote folate oxidation or interconversion among vitamers. Therefore, samples and extracts must be handled under controlled conditions, often with antioxidants or reducing agents incorporated into the extraction buffer [21,61].

Enzymatic deconjugation is essential because most naturally occurring food folates are present as polyglutamates. Folate conjugase is found in human plasma, rat serum, chicken pancreas, and plant sources, and hydrolyzes polyglutamates into shorter-chain or monoglutamate forms. Incomplete hydrolysis may lead to underestimating the folate content, particularly when the analytical method primarily detects monoglutamates. The efficiency depends on the enzyme source and activity, incubation conditions, sample-to-enzyme ratio, and the food matrix [10,62].

For starch- or protein-rich foods, sequential or combined enzyme digestion using α-amylase and protease can improve folate release. Although these treatments increase recovery in certain matrices, they are not universally necessary. Additional enzymatic steps increase sample preparation time and may also contribute to folate losses. Therefore, the use of additional enzymes should be justified based on matrix-specific recovery and validation studies to minimize analytical bias [63,64,65].

Folate extraction generally involves homogenization under controlled temperature and other appropriate conditions. This process facilitates the release of folate from cellular structures, denatures folate-binding proteins, and inactivates endogenous enzymes. Accordingly, buffer composition, pH, temperature, reducing agents, and extraction time should be optimized and reported in sufficient detail to ensure reproducibility. Extracts may also require further purification to remove interfering compounds. Folate-binding protein affinity chromatography provides a selective cleanup approach, while solid-phase extraction and ultrafiltration offer alternative strategies. Recovery studies, matrix-matched controls, and certified reference materials are important for identifying potential losses and evaluating matrix effects during sample preparation.

### 3.4. Measurement of Folates and Interpreting Reported Food Folate Values

The microbiological assay has been used to measure total folate in foods. It typically uses *Lactobacillus rhamnosus*, whose growth reflects the amount of folate available in the assay medium. The method is sensitive, relatively inexpensive, and responsive to several biologically active folate forms, making it suitable for a wide range of foods. However, it has several limitations, including the inability to distinguish individual vitamers. The results may be affected by incomplete extraction or deconjugation, matrix inhibition, differences in microbial response, and food components that promote or suppress bacterial growth [61,62].

High-performance liquid chromatography (HPLC) provides greater chemical specificity by separating individual folate forms before ultraviolet, fluorescence, or electrochemical detection. However, its sensitivity and accuracy may be limited by low concentrations of folates in food, weak ultraviolet absorbance, co-eluting matrix components, and differences in detector response. Additional challenges include the availability, purity, and stability of folate standards [66,67]. Consequently, liquid chromatography–tandem mass spectrometry (LC–MS/MS) has become an important method for folate vitamer profiling because it combines high sensitivity and selectivity and enables the quantification of multiple folate forms in a single analysis [22]. Stable-isotope dilution can further improve accuracy by using labeled analogs to compensate for analyte losses, extraction variability, and instrumental variation [41]. However, LC–MS/MS requires specialized equipment and expertise, well-characterized standards, and careful sample handling to minimize folate degradation and interconversion.

The analytical method selection can influence the reported folate concentration. Microbiological assays and LC–MS/MS may yield different results because of incomplete polyglutamate deconjugation, differences in microbial responses to individual vitamers, unmeasured folate forms, or matrix effects. In relation to methodological discrepancies, the importance of complete deconjugation and broad coverage of folate vitamers has been emphasized [10]. Method validation should address accuracy, recovery, matrix effects, and analyte stability. Certified reference materials and interlaboratory comparisons are valuable for assessing analytical performance. Evidence from diverse food matrices also indicates that deconjugation efficiency depends on both enzyme source and the food matrix, highlighting the need for matrix-specific validation [68].

A comparison table lists the strengths, limitations, vitamer coverage, and most appropriate applications of the analytical methods, as shown in Table 8. Food folate values should be interpreted in relation to analytical method used and the folate forms measured. In microbiological assays, reported total folate reflects the combined biological activity of folate compounds under the specific assay conditions. In chromatographic methods, total folate is generally calculated from the concentrations of individual quantified vitamers, with appropriate conversion based on molecular weight where necessary. In some cases, total folate is reported without accounting for differences among vitamers. Therefore, studies should clearly describe the sample source, basis of expression, extraction and deconjugation procedures, analytical method, vitamers measured, and quality-control results. Folate values obtained using different analytical methods may not be directly comparable unless recovery, calibration, adequate controls, and vitamer coverage are comparable. Natural folates and added folic acid should also be reported separately whenever possible.

## 4. Food Folate Quality from Matrix Release to Bioavailability

### 4.1. From Total Folate Content to Biological Availability

Bioavailability is another key challenge when evaluating dietary folate. Natural food folates are usually less bioavailable than folic acid because they must be released from food matrix, deconjugated by intestinal enzymes, and maintained in reduced forms during absorption. DFEs provide a platform for comparing interconversion: 1 μg DFE is equivalent to 1 μg naturally occurring folate or 0.6 μg folic acid consumed with food [7,11]. However, this conversion assumes relatively consistent bioavailability across food sources, which may not accurately reflect the variability. Clinical studies indicate that folate bioavailability varies according to food source, folate form, reference dose, study design, and biomarker used [5,12]. Therefore, natural food folates, folic acid supplements, and fortified foods should be evaluated separately when estimating biological availability.

Folate quality cannot be determined solely from the measured folate concentration of a food. The reported concentration reflects the analytical method used and does not account for subsequent processes, including digestion, intestinal release, absorption, metabolism, and tissue utilization. Accordingly, folate content, bioaccessibility, absorption, and bioavailability should be considered distinct but interconnected measures. Natural food folates occur in plant or animal tissues as mixtures of vitamers, often predominately in polyglutamate form. Before intestinal absorption, these folates must be released from the food matrix and be hydrolyzed to monoglutamates.

Bioaccessibility refers to the proportion of food folate released from the matrix during digestion and made available for uptake by intestinal cells. Bioavailability is a broader concept that encompasses the processes of release, absorption, metabolism, transport, retention, and utilization. It may be expressed as the proportion of an ingested dose that reaches the systemic circulation or relative to a reference compound, typically folic acid or a defined reduced folate. Bioefficacy, in contrast, describes the capacity of an absorbed folate to support or improve a biological function [8,70,71]. Estimates of the bioavailability of natural food folates vary widely depending on the food matrix, vitamer profile, polyglutamate chain length, administered dose, baseline folate status, study duration, and biomarker selected [5,19]. Therefore, total folate concentration should be regarded as a starting point for evaluating food folate quality rather than as a definitive measure of its nutritional value. To define food folate quality, a summary table presents key determinant factors, including total folate concentration, vitamer profile, polyglutamate distribution, stability during processing, bioaccessibility, absorption, and biological efficacy, along with appropriate analytical methods, as shown in Table 9.

### 4.2. Food-Matrix Determinants of Folate Release

The food matrix is the structural and physicochemical environment in which folates are present. In plant foods, folates are located in the cytosol, mitochondria, vacuoles, and plastids and surrounded by cellular membranes and walls. Their release depends, in part, on the extent to which chewing and digestion disrupt these structures. This effect may vary among leafy vegetables, fruits, grains, seeds, and legumes, depending on tissue structure and cell-wall composition.

Dietary fiber may affect folate release by maintaining cellular structure, increasing intestinal viscosity, or interacting with folates and digestive enzymes. However, its effects are not uniformly inhibitory, and the physical entrapment of folates within intact plant tissues may be more important than total fiber content [8,19]. Other matrix components may also influence folate bioaccessibility. Proteins can bind folates or alter their release during digestion. In milk, folate-binding proteins can protect 5-MTHF from degradation and may delay its availability for absorption. Starch-rich structures can entrap folates within granules or protein–starch networks, particularly in cereals and legumes. Lipids may influence gastric emptying and intestinal transit. Polyphenols, minerals, and organic acids have also been reported to interact with folate or folate-hydrolyzing enzymes, although the available evidence remains unclear [8].

The food matrix may exert both inhibitory and protective effects. Dense cellular structure may limit folate release, whereas binding to proteins or other components may protect reduced folates during digestion. The net effect of the food matrix should be considered in terms of the balance among physical release, chemical stability, enzymatic deconjugation, and intestinal uptake.

Moreover, reduced folates are susceptible to oxidative degradation, and pH changes can affect stability and promote interconversion among vitamers [21]. Interconversion may alter the measured vitamer profile without an equivalent loss of total folate. For example, oxidation can generate products such as MeFox, an oxidation product of 5-MTHF that does not support growth in the microbiological assay [10]. Differences in sample preparation and the compounds detected can therefore contribute to variation among analytical results. Matrix protection and digestion or extraction conditions therefore influence both recovery and interpretation [21].

### 4.3. Gastrointestinal Release, Deconjugation, and Intestinal Absorption

Following ingestion, natural folates must be released from cellular structures and food matrices. Most dietary folates occur as polyglutamates with several γ-linked glutamate residues. These polyglutamates must be hydrolyzed to monoglutamates by glutamate carboxypeptidase II at the brush border of the proximal small intestine before they can be transported across the epithelium [19,77]. The extent of deconjugation depends on polyglutamate chain length, food matrix composition, pH value, and enzyme activity. Incomplete hydrolysis reduces the amount of folate available for intestinal epithelial cells [79].

Following deconjugation, monoglutamate folates are absorbed primarily in the duodenum and proximal jejunum. Dietary folate uptake is primarily mediated mainly by the proton-coupled folate transporter (PCFT), encoded by SLC46A1, which transports both reduced folates and folic acid [77,80]. The essential role of PCFT is demonstrated by hereditary folate malabsorption, in which SLC46A1 mutations cause severe systemic and neurological deficiency. The reduced folate carrier (RFC), encoded by SLC19A1, is widely expressed and mediates folate transport into cellular for utilization in peripheral tissues [80]. Within enterocytes, folates undergo metabolism before being released into the portal circulation, with 5-MTHF e predominating. The extent and pattern of this metabolism depend on folate form and dose. The liver subsequently plays a central role in folate storage, redistribution, metabolism, and enterohepatic circulation [5].

Gastrointestinal and hepatic conditions may interfere with these processes. Celiac disease, inflammatory bowel disorders, intestinal resection, and diseases affecting the proximal small intestine may reduce absorption. Altered pH, brush-border damage, or hepatic dysfunction may also affect folate metabolism. Alcohol disrupts multiple stages of folate absorption and metabolism, while certain medicines may interfere with folate transport or metabolic pathways [1,81]. Physiological and nutritional status also influence folate utilization. Folate requirements increase during pregnancy because of maternal tissue growth and fetal development. Older adults may be at increased risk of inadequate folate status because of reduced dietary intake, chronic disease, medication use, or vitamin B_12_ deficiency, which can impair intracellular utilization of 5-MTHF [24].

### 4.4. Natural Food Folates Versus Synthetic Folic Acid

Natural food folates and synthetic folic acid differ in chemical structure, stability, bioavailability, and metabolic handling. Food folates, commonly present as polyglutamates, are mainly reduced tetrahydrofolate derivatives in plant or animal matrices. These forms are more chemically labile and exhibit greater variation in bioavailability because they require release from matrix and enzymatic deconjugation of polyglutamate chains. In contrast, folic acid (pteroylmonoglutamic acid) is a fully oxidized monoglutamate used in fortified foods and supplements. It is more stable and does not require deconjugation, resulting in more efficient and predictable absorption [1,7]. After absorption, folic acid must be reduced to dihydrofolate by dihydrofolate reductase and subsequently to tetrahydrofolate before entering the reduced-folate pool. Because human dihydrofolate reductase activity is relatively low and varies among individuals, higher doses consumed at once may result in unmetabolized folic acid entering the circulation. The health impact of this exposure remains uncertain [13,82].

These differences are reflected in the dietary folate equivalent (DFE) system. One microgram of DFE corresponds to 1 μg of naturally occurring food folate, 0.6 μg of folic acid consumed with food, or 0.5 μg of folic acid consumed on an empty stomach [11]. Folic acid fortification has improved population folate status and reduced the prevalence of neural-tube defects in many countries. Its stability, low cost, and reliable incorporation into staple foods make it suitable for population-level nutritional interventions [83].

Natural folates and folic acid also differ in metabolic handling. Absorbed natural folates can enter the reduced-folate pool through intracellular interconversion, whereas folic acid must first undergo sequential reduction before it can participate in folate metabolic. Accordingly, supplemental or fortified 5-MTHF has been proposed as an alternative to folic acid because it is already in a reduced form and resembles the major circulating form of folate. Pharmacokinetic studies have shown that 5-MTHF can improve folate biomarkers without producing unmetabolized folic acid [84]. However, evidence remains insufficient to establish that 5-MTHF prevents neural-tube defects as effectively as folic acid.

Increasing attention to the consideration of fortified foods and supplements to total folate intake has raised questions about potential exposure to high amounts of folic acid. These concerns include the presence of circulating unmetabolized folic acid, interactions with vitamin B_12_ deficiency, potential neurological effects, and possible effects on carcinogenesis. Current evidence does not establish that unmetabolized folic acid is harmful, and uncertainty remains regarding its effects at high levels of exposure [13,14]. In an observational study, higher intake of natural food folate was associated with a lower risk of death and end-stage kidney disease among participants with chronic kidney disease, whereas synthetic folic acid from fortified foods did not show the same association. Further analysis using data from the National Health and Nutrition Examination Surveys (NHANES) found that food folate intake and serum 5-MTHF, but not synthetic folic acid, were inversely associated with all-cause mortality. In addition, serum unmetabolized folic acid was associated with higher all-cause mortality [85]. These observational findings should be interpreted cautiously and do not establish causality.

Overall, natural folates, folic acid, and other reduced forms differ in stability, absorption, and metabolism handling. These differences should therefore be considered when applying folate-equivalence systems, assessing dietary folate intake, and designing food-based and folic acid fortification strategies.

## 5. Dietary Folates, Nutritional Status, and Human Health

Adequate folate intake is essential for preventing deficiency, supporting normal hematological function, and promoting health fetal development. Folate status and folic acid supplementation have also been investigated in relation to cardiovascular disease, cognitive decline, metabolic dysfunction, inflammation, and cancer [86,87,88,89,90,91]. Evidence for these outcomes is still developing, whereas the benefits of preventing folate-deficiency anemia and neural-tube defects are well established. Considering supplement dose and duration, alongside differences between natural folate in foods and folic acid from fortification or supplements, may help clarify their relationships with health outcomes.

### 5.1. Assessment of Folate Status

Folate status can be assessed using dietary assessment, circulating biomarkers, functional markers, and hematological indices. Each indicator reflects a different aspect of folate nutrition, and no single measure adequately captures intake, tissue stores, metabolic function, or clinical status. Analytical method, physiological stage, vitamin B_12_ status, renal function, medication use, and recent dietary intake or folic acid supplements should therefore be considered when interpreting folate status [1,92].

Serum or plasma folate reflects recent intake, fortification, or supplementation and is therefore useful for evaluating short-term exposure. A low concentration may indicate inadequate folate intake or a negative folate balance, but a single measurement does not establish persistent folate deficiency. Conversely, recent folic acid supplement may transiently increase serum folate concentrations without reflecting long-term or tissue folate status. Red blood cell (RBC) folate, in contrast, reflects longer-term status because folate is incorporated into erythrocyte during erythrocytes and remains within circulating erythrocytes for approximately their 120-day lifespan. RBC folate is less affected by recent dietary intake and is useful for assessing folate status in women of reproductive age. The World Health Organization (WHO) recommends an RBC folate concentration above 906 nmol/L at the population level to indicate folate status associated with optimal prevention of neural-tube defects; however, this threshold should not be interpreted as an individual diagnostic cutoff [92]. Analytical methods can also influence measured serum and RBC folate concentrations. Microbiological assays, protein-binding assays, and LC–MS/MS methods may yield systematically different results and therefore require appropriate calibration and suitable reference materials [1].

Plasma homocysteine provides a functional but nonspecific indicator of folate status because inadequate folate can impair the remethylation of homocysteine to methionine. Although concentrations often decrease following folate supplementation, they are also influenced by vitamins B_12_, B_6_, and B_2_, as well as renal function, smoking, alcohol consumption, medication use, age, and genetic variation [93]. Therefore, homocysteine should be interpreted together with folate and vitamin B_12_ measurements. Moreover, macrocytosis, hypersegmented neutrophils, and megaloblastic anemia generally occur relatively late in severe or prolonged deficiency and may also be manifestations of vitamin B_12_ deficiency [50,94]. A comprehensive assessment of folate should therefore integrate folate and vitamin B_12_ biomarkers with homocysteine concentration, hematological findings, dietary assessment, and relevant clinical history.

### 5.2. Pregnancy and Early Development

Folate requirements increase during pregnancy because of maternal tissue expansion, placental development, erythropoiesis, and rapid fetal growth. The neural tube closes early in embryonic development, often before pregnancy is recognized. Adequate folate status should be established during the reproduction years. Accumulating evidence indicates that prevention of NTDs is the most well-established benefit of folic acid supplementation. Randomized trials and population-based studies have demonstrated that periconceptional folic acid supplementation reduces the risk of NTDs, including spina bifida and anencephaly [95,96,97].

Three decades after the initial evidence was established, this issue was reassessed through a comprehensive review of expert opinions, observational studies, randomized clinical trials, and systematic reviews published between 1990 and 2021. Updated guidance suggests that oral folic acid supplementation, or dietary folate intake combined with multivitamin or micronutrient supplementation, is associated with lower rates of NTDs, other congenital anomalies, and pregnancy complications [98]. The ECLIPSES study in pregnant women also demonstrated that periconceptional folic acid supplementation significantly reduced folate deficiency and insufficiency [99]. Accordingly, the 2023 U.S. Preventive Services Task Force recommends 400–800 μg/day of folic acid for individuals who are planning to become or capable of becoming pregnant [100]. Nevertheless, because many women of reproductive age do not obtain sufficient folate from their diets, folic acid supplementation and folate-fortified foods remain important strategies. However, concerns have been raised that some women taking folic acid supplements may exceed the recommended tolerable upper intake level of 1000 μg/day. This highlights the need to further evaluate supplementation practices and intake limits to minimize potential health risks [101].

Although folate is important for maternal and fetal health, potential risks associated with both inadequate and excessive intake warrant consideration. A case–control study of congenital heart defects found that both low and excessively high maternal serum folate concentrations during early-to-mid pregnancy were associated with an increased risk of congenital heart defects in offspring [102]. An epidemiological study reported that excessive folic acid supplementation and high maternal folate concentrations were linked to gestational diabetes, child neurodevelopmental outcomes and higher birth weight [103], highlighting the need to better define safe upper limits for folic acid intake and maternal blood folate concentrations. However, systematic reviews and meta-analyses suggest that folic acid supplementation during pregnancy may reduce the risk of autism spectrum disorder, attention-deficit/hyperactivity disorder, and other behavioral problems in offspring [104].

Folate-dependent one-carbon metabolism may also influence fetal programming and childhood development through nucleotide synthesis and DNA methylation. Both inadequate folate intake and excessive exposure to methyl donors during sensitive developmental periods may alter these processes. Although research in this area remains limited, current supplementation guidelines primarily focus on preventing NTDs. Current evidence shows that folate-rich foods support maternal nutrition, and does not support reducing the recommended folic acid intake during the periconceptional period [4,100]. Further studies are needed to clarify the potential long-term effects of high-dose folic acid intake.

Naturally folate-rich foods are also important because they provide fiber and other essential nutrients in addition to folate. However, variations in folate content, stability, and bioavailability make dietary sources less reliable as substitutes for periconceptional folic acid supplementation. Natural food folates must be released from food and hydrolyzed from polyglutamates before absorption. Folic acid does not require deconjugation but undergoes reduction before entering the reduced-folate pool, and supplemental 5-MTHF bypasses this reduction. This difference partially explains why excessive folic acid intake can produce unmetabolized folic acid in the blood. Overall, evidence on the broader effects of abundant food folate intake supports benefits during pregnancy, while evidence on excessive intake remains inconclusive [105].

### 5.3. Hematological, Neurological, and Cognitive Health

Folate is essential for erythropoiesis because red blood cell development requires continuous purine and thymidylate synthesis. Folate deficiency impairs DNA replication while allowing cytoplasmic maturation to continue, resulting in nuclear–cytoplasmic asynchrony and megaloblastic changes. Clinical manifestations include macrocytic anemia, fatigue, weakness, pallor, leukopenia, thrombocytopenia, and hypersegmented neutrophils [3].

Vitamin B_12_ status should be considered alongside folate status because both nutrients are involved in methionine synthase activity and one-carbon metabolism. Folate and vitamin B_12_ deficiencies can cause similar hematological abnormalities; however, vitamin B_12_ deficiency can additionally cause neurological damage. Neurological manifestations include peripheral neuropathy, impaired proprioception, gait disturbances, cognitive changes, and subacute combined degeneration of the spinal cord. Although folate treatment can correct the anemia associated with vitamin B_12_ deficiency, it does not reverse neurological damage and may delay recognition of the underlying vitamin B_12_ deficiency. This interaction is related to the methyl-folate trap. Vitamin B_12_ is required by methionine synthase to transfer the methyl group from 5-MTHF to homocysteine. When vitamin B_12_ is deficient, folate becomes metabolically trapped as 5-MTHF, reducing the availability of tetrahydrofolate derivatives required for nucleotide synthesis, even when circulating folate concentrations are normal or elevated [23,24]. Therefore, vitamin B_12_ status should be evaluated before treating presumed folate-deficiency anemia, particularly in older adults, strict vegetarians, and individuals with malabsorption.

Low folate status and elevated homocysteine concentrations have also been associated with cognitive impairment, dementia, depression, and brain atrophy. Potential mechanisms include impaired methylation and neurotransmitter metabolism, vascular injury, oxidative stress, and homocysteine-related neurotoxicity [106,107]. In the FACIT trial, long-term folic acid supplementation improved selected cognitive outcomes in adults with elevated homocysteine in a setting without mandatory folic acid fortification [108]. The neurological implications of high folate status may also depend on vitamin B_12_ status. Epidemiological studies have reported associations between high serum folate combined with low vitamin B_12_ and anemia, cognitive impairment [109], and altered metabolic biomarkers [24]. However, a meta-analysis involving approximately 22,000 participants found that homocysteine-lowering B-vitamin supplementation did not significantly influence cognitive aging. Potential benefits were observed primarily among individuals with low folate status, elevated homocysteine, adequate vitamin B_12_ status, or early cognitive impairment [15]. These relationships remain unclear; the findings support assessing folate and vitamin B_12_ status together rather than assuming higher folate intake is universally beneficial.

### 5.4. Cardiovascular Health and Metabolic Disorders

Folate has been extensively studied in cardiovascular health because it supports homocysteine remethylation. Folate deficiency promotes homocysteine accumulation, disturbed methylation, mitochondrial dysfunction, and impaired nucleotide repair [23,27]. Increased homocysteine is associated with endothelial dysfunction, oxidative stress, inflammation, thrombosis, and cardiovascular risk [110]. Folic acid and other B vitamins help reduce homocysteine, particularly in populations with low folate status. However, improvement in this biomarker does not necessarily reduce clinical events. In the China Stroke Primary Prevention Trial, enalapril combined with folic acid reduced primary stroke risk compared with enalapril alone in hypertensive adults without folic acid fortification [111]. A meta-analysis of randomized trials showed little or no effect of homocysteine-lowering B vitamins on myocardial infarction, cardiovascular disease, cancer, or all-cause mortality [15]. Clinical trials are heterogeneous; benefits may be clearer when baseline folate status is relatively low, homocysteine is elevated, or participants have existing metabolic disease.

Beyond cardiovascular disease, folate has also been linked to obesity, insulin resistance, diabetes, and metabolic syndromes [112]. Folate-driven one-carbon metabolism may also influence glucose regulation, hepatic methylation, lipid metabolism, mitochondrial function, and endothelial health. Laboratory studies have demonstrated that folate deficiency increased body weight in high-fat-fed mice and promoted lipid accumulation and leptin production in adipocytes, indicating that inadequate folate status may be a risk factor for adiposity [113]. Similarly, dietary folic acid supplementation reduced adipose accumulation in broiler chicken through inhibiting adipocyte proliferation and differentiation and mediating gut microbiota changes and short-chain fatty acid production [114]. An animal study showed that mice fed a high-fat high-fructose diet for one year developed renal inflammation and fibrosis through the raised phosphorylation of STAT3 and Smad2/3. Folate deficiency increased serum homocysteine and worsened the pathologic process in mice, highlighting that inadequate folate status exacerbates inflammation and enhances renal fibrosis [115].

A cross-sectional study of US adults from the 2011–2018 NHANES showed a negative correlation of serum folate with nonalcoholic fatty liver disease and advanced liver fibrosis [116]. A cohort study using the UK Biobank database shows that moderate folate intake from food about 300 µg may reduce the risk of metabolic dysfunction-associated steatotic liver disease, suggesting that folate-rich diets may be a viable strategy for the prevention of metabolic disorders [90]. Meta-analyses reported that folic acid supplementation modestly improved fasting glucose, insulin, or insulin resistance, but the data remained inconsistent for glycated hemoglobin and diabetes incidence [112,117]. According to current evidence, the data do not support folic acid as an independent treatment for diabetes or metabolic syndrome [117].

A systematic review showed that high folate exposure cannot correct the metabolic disorder caused by vitamin B_12_ deficiency, which is associated with adverse metabolic outcomes in mothers or offspring [118]. In addition, folate levels in red blood cells and homocysteine are linked to HOMA-IR in people with severe obesity. A cohort study has challenged the benefits of folic acid-fortified foods for this group, especially for those who are low in vitamin B_12_. Age and RBC folate were independently associated with parameters of metabolic syndrome [119]. This highlights the need to balance one-carbon nutrition rather than supply folate alone.

Clinical studies of inflammatory and immune outcomes are limited and difficult to explain because folate-rich foods such as vegetables, legumes, and fruits always contain multiple bioactive compounds. Similarly, supplementation trials often combine folic acid with vitamins B_6_ and B_12_. Adequate folate is necessary for normal vascular, metabolic, redox, and immune function, but supplementation above adequacy has not consistently prevented cardiometabolic or inflammatory disease [120,121].

### 5.5. Inflammation and Immune Regulation

A growing body of evidence indicates that folate plays an important role in immune regulation. Immune cell proliferation depends on folate-mediated nucleotide and protein synthesis, and folate deficiency can impair lymphocyte proliferation and cell-mediated immunity [122]. In folate-deficient cells, folate or nucleoside repletion rapidly restored T-cell proliferation, reduced DNA uracil accumulation, and increased the CD4^+^/CD8^+^ ratio. These findings suggest that folate status influences immune regulation, particularly by affecting CD8^+^ T-cell proliferation during folate insufficiency [123]. A population-based MEIA study of 594 participants in Germany found that higher folate concentrations were associated with greater proportions of effector memory and certain CD8^+^ T-cell subsets, but lower proportions of naïve and central memory CD8^+^ T cells; however, these associations were not statistically significant [124]. Although preliminary, these findings suggest a potential relationship between serum folate status and specific T-cell subsets, particularly CD8^+^ populations. Further interventional studies are needed to establish whether folate status directly influences immune function.

Monocytes and macrophages are important phagocytes in immune defense and express the beta isoform of the folate receptor (FRβ), which binds folate under conditions involving suppressive cytokine exposure. An in vitro study showed that FRβ blockade or deletion impaired the ability of macrophages to suppress T-cell activity, suggesting that FRβ may act as a regulatory component of the tumor immune response. Conversely, higher FRβ expression has been negatively associated with survival in human cancers, supporting a potential role in tumor immunosuppression [125]. In addition, a laboratory study of splenic-derived dendritic cells found that folate deficiency reduced MHC II and CD80 expression and impaired dendritic-cell-induced T-helper (Th) cell differentiation and cytokine production. These findings suggest that folate may contribute to Th-cell homeostasis [126].

Folate deficiency can promote homocysteine accumulation [23,27], which has been associated with increased oxidative stress and inflammation [110]. Homocysteine has been investigated as a biomarker of disease risk and progression, although its clinical relevance varies among conditions. It has also been linked to immune cell function and differentiation, including those of T cells, B cells, and macrophages. These findings have increased interest in targeting one-carbon metabolism as a potential strategy for modulating immune-related diseases. However, further research is needed to clarify the mechanisms involved and determine whether interventions targeting one-carbon metabolism or related metabolites can improve disease treatment and monitoring [127].

### 5.6. Folate and Cancer

Folate has a dual role in cancer biology. Adequate folate status supports DNA synthesis and normal DNA methylation, whereas deficiency can cause uracil misincorporation, chromosome breakage, genomic instability, and abnormal DNA methylation, potentially contributing to carcinogenesis. However, premalignant and malignant cells also require folate for rapid nucleotide synthesis. Consequently, abundant folate after neoplastic initiation may support tumor growth [128]. This dual role suggests that folate may be protective before neoplastic lesions develop but have neutral or potentially adverse effects after lesions are established. Its effects may depend on dose, timing, baseline folate status, tissue type, genetic background, and whether folate is obtained from natural foods or folic acid supplements [129,130]. Therefore, cancer stage, exposure source, dosage, and duration should be specified.

Evidence regarding colorectal cancer has been extensively studied but remains inconclusive. Because the intestinal epithelium undergoes rapid cell turnover, it may be particularly sensitive to changes in folate status. A systematic review and meta-analysis of observational studies, including 24 cohort studies involving 6165,894 individuals and 37,280 colorectal cancer cases, found that higher dietary folate intake was associated with a lower risk of colon cancer, particularly among populations with moderate or high alcohol consumption, but not among non-drinkers [131,132]. However, folate-rich foods also contain fiber, polyphenols, carotenoids, and other potentially protective components, making it difficult to isolate the specific contribution of folate [133]. In contrast, folic acid supplementation at 1000 μg/day in individuals with previous colorectal adenomas did not prevent adenoma recurrence during the initial three-year surveillance period. At the second examination, conducted three or five years later, the folic acid group had more advanced and multiple adenomas [134]. These findings raise concerns about supplementation after a history of neoplasia and should not be generalized to habitual intake of folate-rich foods.

Baseline status may also modify response, although evidence is inconsistent. In a separate randomized trial, 1000 μg/day of folic acid was associated with fewer recurrent adenomas among participants with baseline plasma folate ≤ 7.5 ng/mL, but not among those with higher concentrations [135]. This study-specific cutoff should not be interpreted as a universal deficiency threshold or an indication for supplementation. Moreover, the earlier adenoma trial found no significant interaction with baseline plasma folate [134]. An animal study further suggested that folate supplementation may promote colorectal cancer progression and immune suppression by impairing CD8^+^ T-cell recruitment and antitumor activity, potentially facilitating immune evasion [136].

Other studies have reported divergent findings. Zhu and colleagues developed a machine-learning-based folate score and identified CYP26A1 as a potential folate metabolism-associated oncogene in colorectal cancer [137]. Meta-analyses of randomized controlled trials, however, have generally found no significant effect of folic acid supplementation on overall cancer incidence or major site-specific cancers during 5.2-year follow-up [138,139]. One randomized trial reported an increased incidence of prostate cancer among participants receiving 1000 μg/day of folic acid supplementation, although this finding was not consistently observed in individuals with higher baseline dietary folate intake [140]. In two Norwegian cardiovascular trials, 800 μg/day of folic acid combined with 400 μg/day of vitamin B_12_ was associated with increased cancer incidence and mortality after a median 39 months of treatment and 38 additional months of follow-up [141]. Evidence for breast, pancreatic, lung, and other cancers remains heterogeneous and may vary according to lifestyle, baseline nutritional status, and supplement exposure.

Observational dose–response analyses have associated each additional 100 μg/day of dietary folate with approximately 4.3% lower head-and-neck squamous cell carcinoma risk and 7% lower pancreatic cancer risk [128]. These estimates describe associations within the studied intake ranges, not the effects of prescribing additional folate. For breast cancer, one prospective-study meta-analysis suggested lower risk at 200–320 μg/day and higher risk above 400 μg/day. However, these findings still cannot establish 400 μg/day as a carcinogenic threshold [128].

Another laboratory study hints that folate is positively associated with cancer growth. Folate receptor alpha (FRα) plays an important role in cellular folate uptake and metabolism and is frequently overexpressed in several cancer types. Consequently, FRα has been investigated as a biomarker for tumor identification, prognosis, and chemotherapy resistance. It has also been targeted in antibody–drug conjugates, small-molecule drug conjugates, and chimeric antigen receptor (CAR) T-cell approaches, making it a potential target in precision oncology [142].

Overall, the evidence should distinguish among folate deficiency, naturally occurring food folates, folic acid from fortified foods, and high-dose folic acid supplementation. Adequate dietary folate supports genomic maintenance, but current evidence does not support the use of high-dose folic acid for cancer prevention. At present, available evidence does not establish that recommended folic acid intake causes cancer. Further long-term studies with well-characterized baseline folate status, lesion stage, dose, duration, and chemical form are needed to clarify the relationship between folate exposure and cancer development and progression.

### 5.7. Risks Associated with Excessive Folic Acid Exposure

Natural food folates have not been associated with adverse effects at usual dietary intakes. Safety concerns primarily relate to folic acid from fortified foods and supplements because synthetic folic acid is more stable and generally more bioavailable than naturally occurring food folates. Tolerable upper intake levels therefore apply primarily to synthetic folic acid and supplemental folate forms rather than naturally occurring food folates [11,14].

A key concern is that high folic acid intake can mask the megaloblastic anemia caused by vitamin B_12_ deficiency, possibly allowing neurological damage to go unnoticed. Observational studies have linked high folate and low B_12_ status to cognitive impairment, anemia, and metabolic changes. However, these associations may be influenced by other factors, and their clinical significance is still unclear [24,109].

Another concern is the presence of unmetabolized folic acid in the circulation. Folic acid must be reduced by dihydrofolate reductase before entering the reduced-folate pool. Because dihydrofolate reductase activity is relatively low and variable in humans, folic acid intake exceeding metabolic capacity may result in detectable unmetabolized folic acid in the circulation. This phenomenon has been reported particularly among populations exposed to folic acid through fortified foods and supplements [13,143]. Unmetabolized folic acid has been investigated in relation to natural killer cell activity, immune regulation, cognition, and cancer development. The NIH workshop concluded that although observational and experimental findings raise potential concerns, the evidence remains inconsistent and does not establish specific clinical harm at recommended intake levels [13]. A recent review reported that higher folic acid intake may increase circulating unmetabolized folic acid and influence inflammation, angiogenesis, and neurotoxicity; however, these proposed effects require further confirmation [144].

Regarding folic acid safety, the most established clinical concern is the potential masking of undiagnosed vitamin B_12_ deficiency, which may allow neurological damage to progress. Excessive unmetabolized folic acid exposure in cancer risk remains biologically plausible but has not been clearly demonstrated. The 2023 EFSA review supports the current tolerable upper intake level at 1000 μg/day for adults, including pregnant and lactating women, covering the combined intake of folic acid and authorized 5-MTHF calcium and glucosamine salts from fortified foods and supplements. This limit excludes naturally occurring food folates while acknowledging areas of uncertainty [14]. Strategies should emphasize choosing appropriate supplement doses, monitoring vitamin B_12_ in high-risk groups, considering total intake from all sources, and encouraging long-term research to understand better the differences between natural food folates and supplemental folic acid.

## 6. Public-Health Applications

### 6.1. Food-Based Strategies to Improve Folate Status

A practical approach to improving folate status is to promote meals based on a diverse range of local and affordable foods naturally rich in folate, including dark-green vegetables, legumes, fruits, whole grains, nuts, seeds, eggs, and selected animal-source foods. Even foods with moderate folate concentrations can make meaningful contributions when consumed regularly. Liver is an excellent source of folate; however, because of its high vitamin A content, it should be consumed cautiously during pregnancy [145]. Although naturally occurring food folates support long-term nutritional adequacy, they cannot replace periconceptional folic acid supplementation, which remains the most reliable strategy for preventing NTDs [4,100]. Where dietary diversity is limited, biofortification may increase the folate content of staple foods [58,146,147,148]. The effectiveness of these strategies should be evaluated using both biomarkers and dietary assessments. Serum folate primarily reflects recent intake, whereas red blood cell folate provides a better indicator of longer-term folate status. Vitamin B_12_ status should also be monitored, particularly in older adults and populations with low consumption of animal-source foods.

### 6.2. Fortification Benefits and Safety Considerations

Folic acid fortification of widely consumed staple foods, including wheat flour, maize flour, and rice, can effectively improve folate status at the population level. Accordingly, the World Health Organization recommends fortifying staple cereal products with folic acid to help prevent NTDs [149,150]. Mandatory fortification programs generally achieve broader population coverage than voluntary approaches and have been associated with increased serum and red blood cell folate concentrations and reduced rates of NTDs [151,152,153]. However, fortification does not replace periconceptional supplementation or a folate-rich diet because fortified foods may not provide sufficient folic acid to meet the needs of all individuals [100]. Safety considerations primarily concern prolonged or excessive folic acid exposure, particularly its potential to mask vitamin B_12_ deficiency and contribute to unmetabolized folic acid exposure and other potential neurological or cancer-related effects [13,14]. Effective folate policies should therefore integrate dietary diversification, appropriate supplementation, and carefully monitored fortification rather than relying on any single strategy.

### 6.3. Priority Research Questions

Future research should move beyond total folate concentration to evaluate folate forms and their relationships with health outcomes. Longitudinal studies are needed to assess cumulative folic acid exposure from mandatory and voluntary fortification and supplementation, including interactions with vitamin B_12_ status, unmetabolized folic acid, neurological outcomes, and cancer risk. Particular attention should be given to children, older adults, individuals with renal impairment, and high-dose supplement users. Food-composition databases should distinguish total folate, individual folate vitamers, and added folic acid using validated analytical methods. Linking these data with serum and red blood cell folate, homocysteine, and vitamin B_12_ measurements would help clarify how accurately dietary folate estimates reflect physiological exposure. Collaboration among food science, nutrition, clinical research, and public health disciplines will be essential for refining dietary recommendations and developing fortification policies that preserve the established benefits of folate while minimizing unnecessary exposure.

## 7. Conclusions

**Figure 1:**
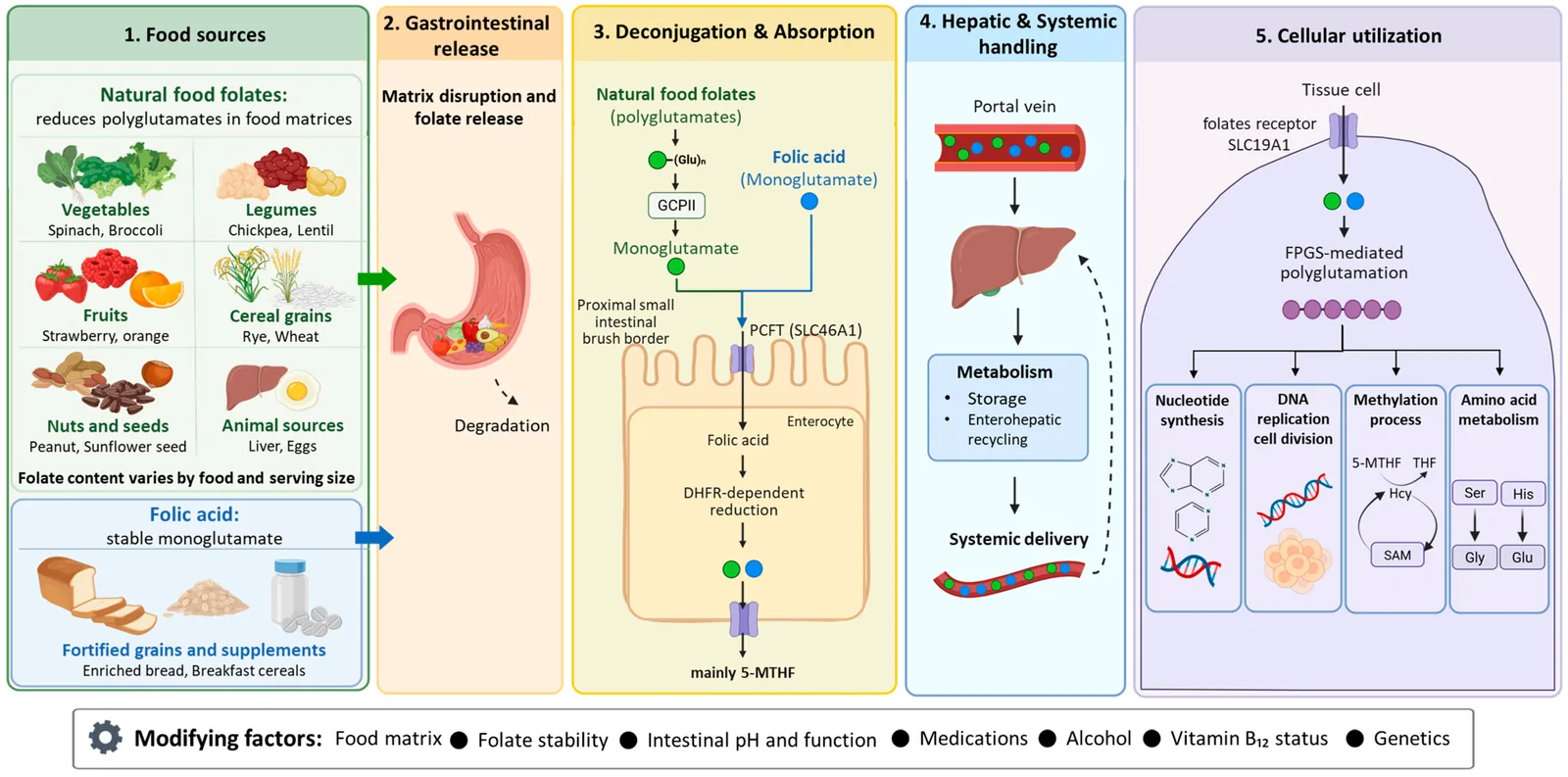
Dietary sources, intestinal absorption, and metabolic utilization of folate. Natural food folates are abundant in leafy vegetables, legumes, fruits, nuts and seeds, and some animal foods, whereas fortified foods and supplements provide additional folate, commonly as folic acid. Natural food folates occur mainly as reduced polyglutamates and must be released during gastrointestinal digestion. Polyglutamates must be hydrolyzed by glutamate carboxypeptidase II (GCPII) as monoglutamates to be absorbed at the proximal intestinal brush border. Folic acid from fortified foods and supplements is already monoglutamylated and bypasses this step. Both forms are taken up primarily through the proton-coupled folate transporter (PCFT; SLC46A1). In enterocytes, folic acid requires dihydrofolate reductase (DHFR)-dependent reduction; reduced-form folates then can enter portal circulation, mainly as 5-methyltetrahydrofolate (5-MTHF). In the body, the liver supports folate metabolism, storage, redistribution, and enterohepatic recycling. Following systemic delivery and cellular uptake through the reduced folate carrier (SLC19A1) or folate receptors, folylpolyglutamate synthetase (FPGS) restores polyglutamation. In cells, folate supports nucleotide synthesis, homocysteine remethylation, S-adenosylmethionine production, DNA synthesis, methylation, and cell proliferation. However, some modifying factors can interfere with these processes, including food matrix, folate stability, intestinal physiology, medications, alcohol exposure, vitamin B12 status, and genetic variation. This figure was drawn using. BioRender Graphing (BioRender, Toronto, ON, Canada; https://www.biorender.com, accessed on 11 September 2026).

Food folate quality depends on both folate content and its availability for physiological use. The framework presented in this review links folate concentration and vitamer composition to analytical reliability, digestive release, bioaccessibility, absorption, and biological efficacy (Figure 1), providing a basis for interpreting differences among food sources and study findings. Folate bioavailability depends on the food matrix, digestive release, deconjugation, absorption, and host metabolism. In contrast, folic acid is stable, highly bioavailable, and well established for the prevention of neural-tube defects.

Adequate folate status clearly prevents folate-deficiency megaloblastic anemia and supports maternal and fetal health, but evidence for additional cardiovascular, cognitive, inflammatory, immune, and cancer-related outcomes remains heterogeneous and inconclusive. These outcomes should be interpreted in relation to chemical form, dose, duration, and baseline nutritional status, including vitamin B12 status.

Public-health strategies should therefore emphasize diverse diets with folate-rich foods, appropriately monitored food fortification, and targeted folic acid supplementation when indicated, particularly when food alone cannot reliably meet requirements. Connecting reliable food-composition data with human bioavailability and health outcomes will help refine this framework and strengthen dietary guidance.

## Figures and Tables

**Table 1:** Folate contents in selected vegetables from different geographical regions ^1^.

Food Source	Folate ^2^ (µg/100 g)		Geographical Origin	Method ^3^	Survey Program, Database, or Study	Ref.
Spinach (*Spinacia oleracea*)	210		Japan	–	Standard Tables of Food Composition in Japan	[28]
	110	(cooked)				
	194		United States	–	USDA FoodData Central	[29]
	194	(dried)	South Africa Egypt	– HPLC	Regional analytical studies	[30]
	129	(dried)				
Spinach	177		Italy	LC-MS	Cultivar and production-season study	[31]
	65–164		Taiwan	Microbio	Market-sampling study	[32]
Spinach (*S. oleracea*), germplasm accessions	54–173		China	LC	Germplasm evaluation studies	[33]
Broccoli (*B. oleracea* var. *capitata* L.)	220		Japan	–	Standard Tables of Food Composition in Japan	[28]
	32–62		Taiwan	Microbio	Market-sampling study	[32]
	63		United States	–	USDA FoodData Central	[29]
Pak choi (*Brassica rapa* subsp. *chinensis*)	146		China	LC	Analytical survey of Chinese vegetables	[34]
Lettuce	136		United States	–	USDA FoodData Central	[29]
Lettuce (*Lactuca sativa*), different cultivars	73–125		Denmark	LC-MS	Cultivar and production-season studies	[31]
Lettuce (*L. sativa*), different cultivars	48–112		Sweden	LC-MS		[31]
Lettuce (*L. sativa*), Iceberg	30–38		Germany	LC-MS		[31]
Lettuce	64		Taiwan	Microbio	Market-sampling study	[32]
Lettuce (*L. sativa*)	80	(dried)	Egypt	HPLC	Market-sampling study	[30]
Water convolvulus (*Ipomoea aquatica*)	120		Japan	–	Standard Tables of Food Composition in Japan	[28]
	55	(cooked)				
	57		United States	–	USDA FoodData Central	[29]
	35	(cooked)				
African lettuce (*Launaea taraxacifolia*)	91		Nigeria	HPLC	Field-sampling studies	[30]
Amaranth (*Amaranthus gangeticus. Linn.)*	81		Taiwan	Microbio	Market-sampling study	[32]
Amaranth (*A.* spp.)	72–130		South Africa	Microbio	Field-sampling studies	[30]
Amaranth (*A. hybridus*)	62		Nigeria	HPLC		[30]
Cowpea (*Vigna unguiculata*)	105–154		South Africa	Microbio	Field-sampling studies	[30]
	141	(dried)		–	–	
Chinese cabbage (*Brassica rapa subsp. chinensis*)	92		South Africa	Microbio	Field-sampling studies	[30]
	84		Taiwan	Microbio	Market-sampling study	[32]
Black nightshade (*Solanum retroflexum*)	56		South Africa	Microbio	Field-sampling study	[30]
Black nightshade (*S. nigrum*)	404	(dried)	Côte d’Ivoire	–	Regional analytical study	[30]
Okra fruit (*Abelmoschus esculentus*)	106		Nigeria	HPLC	Field-sampling study	[35]
Asparagus (*Asparagus officinalis*)	52		United States	–	USDA FoodData Central	[29]
	149	(cooked)				
	191	(frozen)				
Pumpkin (*Cucurbita maxima*)	47	(raw leaves)	South Africa	Microbio	Field-sampling study	[30]
Potato (*Solanum tuberosum*)	23		Finland	HPLC	Market-sampling study	[36]
	10		Egypt	HPLC		[37]
Sweet potato (*Ipomoea batatas*)	22		Egypt	HPLC	Market-sampling study	[37]
Sweet potato (*I. batatas*)	80	(dry leaves)	South Africa	–	Regional analytical study	[30]
Carrot (*Daucus carota*)	21		Egypt	HPLC	Market-sampling study	[37]
	16		Finland	HPLC		[36]
Cucumber (*Cucumis sativus*)	17		Taiwan	Microbio	Market-sampling study	[32]
	14		Egypt	HPLC	Market-sampling study	[37]
	9		Finland	HPLC		[36]
Onion (*Allium cepa*)	12		Egypt	HPLC	Market-sampling study	[37]
	11		Finland	HPLC		[36]
Tomato (*Lycopersicon esculentum*)	11		Egypt	HPLC	Market-sampling study	[37]
	8		Finland	HPLC		[36]

^1^ Approximate values represent selected records from national databases; ranges represent variation among cultivars, samples, locations, or preparation methods. ^2^ Values are expressed as total folate in µg/100 g of edible food unless otherwise specified. Values reported for raw, cooked, or dried foods should not be compared without considering moisture and processing. Added folic acid in fortified products is excluded wherever it could be distinguished from natural folate. ^3^ Analytical methods: LC, liquid chromatography; HPLC, high-performance liquid chromatography; LC-MS, liquid chromatography–mass spectrometry; UPLC–MS/MS, ultra-performance liquid chromatography–tandem mass spectrometry; Microbio, microbiological assay.

**Table 2:** The folate contents of commonly consumed mushrooms in Taiwan.

Mushroom Type	Folate ^1^ (µg/100 g Raw Sample)			Published Database		
				Taiwan ^2^	Japan ^3^	US ^4^
*Lentinus sajor-caju* (funnel woodcap)	98	±	25	22	100	–
*Pleurotus ostreatus* (oyster mushroom)		–		22	92	63
*Pleurotus eryngii,* (king oyster mushroom)		–		54	80	–
*Flammulina filiformis* (enokitake)	50	±	19	20–25	75	–
*Volvariella volvacea* (straw mushroom)		–		58	–	–
*Lentinula edodes* (shiitake)	37	±	20	49	49	32
*Agaricus bisporus* (common mushroom)		–		17–28	28	–
*Hypsizygus tessulatus* (brown beech mushroom)	86	±	8	30	29	–
*Hypsizygus tessulatus* (white beech mushroom)	87	±	10	27	–	–
*Agrocybe aegerita* (poplar mushroom)		–		47	33	–
*Pleurotus cystidiosus* (Abalone mushroom)		–		14	65	–

^1^ Values represent the edible portion of 100 g of raw mushroom sample, analyzed by *Lactobacillus casei* (ATCC 7469) microbiological assay as previously described [39]. Each mushroom was sampled twice at different times and locations. Data are presented as mean ± SD (*n* = 2 independent experiments). ^2^ Taiwan Food Composition Database by Taiwan Food and Drug Administration, Ministry of Health and Welfare, https://consumer.fda.gov.tw/Food/TFND.aspx?nodeID=178 (accessed on 18 October 2026). Folate contents were analyzed by *Enterococcus hirae* ATCC 8043 microbiological assay. ^3^ Standard Tables of Food Composition in Japan—8th edition (Japan, 2023), https://fooddb.mext.go.jp (accessed on 18 October 2026). ^4^ USDA Nutrition Database, https://fdc.nal.usda.gov/ (accessed on 18 October 2026).

**Table 3:** Folate contents in individual fruits and berries from different geographical regions ^1^.

Food Source	Folate ^2^ (µg/100 g)		Geographical Origin	Method ^3^	Survey Program, Database, or Study	Ref.
Strawberry (*Fragaria × ananassa*)	115		Germany	LC-MS/MS	Stable-isotope dilution LC–MS/MS study	[41]
	107	(frozen)				
	98	(lyophilized)				
Strawberry (*F. × ananassa*)	90		Japan	–	Standard Tables of Food Composition in Japan	[28]
	4	(dried)				
Strawberry (*F. × ananassa*)	80		Egypt	HPLC	Market-sampling study	[37]
Strawberry (*F. × ananassa*)	74		Sweden	RPBA	Cultivar, maturity, and harvest-year study	[42,43]
	335–644	(dried)				
Strawberry (*F. × ananassa*)	36		Finland	HPLC	Market-sampling study	[36]
Strawberry (*F. × ananassa*)	24		United States	–	USDA FoodData Central	[29]
Strawberry (*F. × ananassa*)	22		Brazil	–	Brazilian Food Composition Table	[44]
Rose hip (*Rosa* spp.)	96		Sweden	RPBA	Berry-composition survey	[42]
Avocado (*Persea americana*)	83		Japan	–	Standard Tables of Food Composition in Japan	[28]
Avocado (*P. americana*)	81		United States	–	USDA FoodData Central	[29]
Avocado (*P. americana*)	42		Brazil	–	Brazilian Food Composition Table	[44]
Raspberry (*Rubus idaeus*)	46		Sweden	RPBA	Berry-composition survey	[42]
Raspberry (*R. idaeus*)	38		Japan	–	Standard Tables of Food Composition in Japan	[28]
Sea buckthorn (*Hippophae rhamnoides*)	39		Sweden	RPBA	Berry-composition survey	[42]
Orange (*Citrus sinensis*)	34		Japan	–	Standard Tables of Food Composition in Japan	[28]
Orange (*C. sinensis*)	30	(with peel)	United States	–	USDA FoodData Central	[29]
Orange (*C.* spp.)	28		Brazil	–	Brazilian Food Composition Table	[44]
Tangerines (*C. reticulata*)	16		United States	–	USDA FoodData Central	[29]
Blackcurrant (*Ribes nigrum*)	17		Sweden	RPBA	Berry-composition survey	[42]
Blueberry (*Vaccinium* spp.)	11		Sweden	RPBA		[42]
Banana (*Musa paradisiacal*)	13		Finland	HPLC	Market-sampling study	[36]
Banana (*M. paradisiacal*)	8		Egypt	HPLC		[37]

^1^ Approximate values represent selected records from national databases; ranges represent variation among cultivars, samples, locations, or preparation methods. ^2^ Values are expressed as total folate in µg/100 g of edible food unless otherwise specified. Values reported for raw, frozen, or dried foods should not be compared without considering moisture and processing. ^3^ Analytical methods: HPLC, high-performance liquid chromatography; LC-MS/MS, liquid chromatography–tandem mass spectrometry; RPBA, radioprotein-binding assay.

**Table 4:** The folate contents in commonly consumed fruits in Taiwan.

Fruit Type	Folate Contents ^1^ (µg/100 g)			Folate Density (µg/60 kcal)	Published Database		
					Taiwan ^2^	Japan ^3^	US ^4^
Durian	225	±	53	122	138	150	36
Atemoya	95	±	25	56	17	23	–
Strawberry	94	±	13	153	83	90	24
Cherry tomato	66	±	2	123	–	35	–
Kyoho grape	47	±	9	42	4	4	–
Papaya	44	±	10	80	47	44	37
Golden kiwifruit	40	±	10	41	4	32	31
Orange	37	±	3	51	8	34	30
Kiwifruit	30	±	1	32	31	36	25
Pineapple	30	±	6	36	11	11	18
Guava	30	±	7	51	55	41	49
Milk jujube	30	±	0	33	7	19	–
Grapefruit	24	±	3	36	4	15	10
Dragon fruit	23	±	13	26	14	44	–
Lemon	20	±	7	41	7	31	11
Mandarin	18	±	9	25	6	13-24	16
Melon	18	±	5	32	15	32	19
Wax apple	11	±	4	17	24	–	–
Peach	7	±	3	11	6	5	4
Cantaloupe	7	±	5	13	–	–	21
Fuji apple	5	±	4	5	3	5	3
Star fruit	5	±	2	10	46	11	12
Banana	4	±	1	2	16	26	20
Honey jujube	4	±	1	4	7	–	–
Watermelon	2	±	6	3	5	3	3

^1^ Values represent the edible portion of 100 g of fruit, excluding the peel, stem, and other inedible portions, analyzed by *Lactobacillus casei* (ATCC 7469) microbiological assay as previously described [39]. Each fruit was sampled three times at different times and locations. Data are presented as mean ± SD (*n* = 3), except for durian and milk jujube (*n* = 2). The data have been previously published [45]. ^2^ Taiwan Food Composition Database by Taiwan Food and Drug Administration, Ministry of Health and Welfare, https://consumer.fda.gov.tw/Food/TFND.aspx?nodeID=178 (accessed on 18 October 2026). Folate contents were analyzed by *Enterococcus hirae* ATCC 8043 microbiological assay. ^3^ Standard Tables of Food Composition in Japan-8th edition (Japan, 2023), https://fooddb.mext.go.jp (accessed on 18 October 2026). ^4^ USDA Nutrition Database, https://fdc.nal.usda.gov/ (accessed on 18 October 2026).

**Table 5:** Folate contents in individual legumes and pulses from different geographical regions ^1^.

Food Source	Folate ^2^ (µg/100 g)		Geographical Origin	Method ^3^	Survey Program, Database, or Study	Ref.
Chickpea (*Cicer arietinum*)	557		United States	–	USDA FoodData Central	[29]
	172	(cooked)				
Chickpea (*C. arietinum*)	539		Brazil	–	Brazilian Food Composition Table	[44]
	134	(cooked)				
Chickpea (*C. arietinum*)	351–589	(dried)	Saskatchewan, Canada	UPLC–MS/MS	Genotype and location survey	[48]
Chickpea (*C. arietinum*)	148	(dried)	Egypt	HPLC	Market-sampling study	[37]
Pinto bean (*P. vulgaris*)	503		Brazil	–	Brazilian Food Composition Table	[44]
	102	(cooked)				
Lentil (*Lens culinaris*)	479		United States	–	USDA FoodData Central	[29]
	181	(cooked)				
Lentil (*L. culinaris*)	136–182	(dried)	Saskatchewan, Canada	UPLC–MS/MS	Genotype and location survey	[38]
Lentil (*L. culinaris*)	75	(dried)	Egypt	HPLC	Market-sampling study	[37]
Black bean (*P. vulgaris*)	444		United States	–	USDA FoodData Central	[29]
	149	(cooked)				
Common bean (*P. vulgaris*)	165–232	(dried)	Saskatchewan, Canada	UPLC–MS/MS	Genotype and location survey	[48]
Kidney bean (*P. vulgaris*)	96	(dried)	Egypt	HPLC	Market-sampling study	[37]
Edamame (*Glycine max*)	320		Japan	–	Standard Tables of Food Composition in Japan	[28]
	260	(cooked)				
	310	(frozen)				
Soybean (*G. max*), ungerminated seeds	203–231		Bangladesh	LC	Cultivar and germination study	[49]
Natto, fermented soybean (*G. max*)	120		Japan	–	Standard Tables of Food Composition in Japan	[28]
Faba bean (*Vicia faba*)	96	(dried)	Egypt	HPLC	Market-sampling study	[37]
Green pea (*Pisum sativum*)	59	(frozen)	Finland	HPLC	Market-sampling study	[36]
Green pea (*P. sativum*)	52	(dried)	Egypt	HPLC		[37]
Green-bean pods (*Phaseolus vulgaris*)	24–32		Brazil	–	Brazilian Food Composition Table	[44]
	19	(cooked)				
Pea	23–30	(dried)	Saskatchewan, Canada	UPLC–MS/MS	Genotype and location survey	[46]

^1^ Approximate values represent selected records from national databases; ranges represent variation among cultivars, samples, locations, or preparation methods. ^2^ Values are expressed as total folate in µg/100 g of edible food unless otherwise specified. Values reported for raw, flour, or dried foods should not be compared without considering moisture and processing. ^3^ Analytical methods: UPLC–MS/MS, ultra-performance liquid chromatography–tandem mass spectrometry; LC, liquid chromatography; HPLC, high-performance liquid chromatography.

**Table 6:** Folate contents in individual grains and nuts from different geographical regions ^1^.

Food Source	Folate ^2^ (µg/100 g)		Geographical Origin	Method ^3^	Survey Program, Database, or Study	Ref.
**Cereal grains**						
Quinoa (*Chenopodium quinoa*)	190		Japan	–	Standard Tables of Food Composition in Japan	[28]
	184		Brazil	–	Brazilian Food Composition Table	[44]
	184	(dried)	United States	–	USDA FoodData Central	[29]
Oat (*Avena sativa*)	56		Brazil	–	Brazilian Food Composition Table	[44]
Oat	52		United States	–	USDA FoodData Central	[29]
Rye (*Secale cereale*)	31–39		Sweden	HPLC	Cultivar and germination study	[50]
	38		Brazil	–	Brazilian Food Composition Table	[44]
	38	(dried)	United States	–	USDA FoodData Central	[29]
Wheat (*Triticum* spp.)	41		Brazil	–	Brazilian Food Composition Table	[44]
Wheat (*T. aestivum*)	38		United States	–	USDA FoodData Central	[29]
	44	(flour)				
	38		Japan	–	Standard Tables of Food Composition in Japan	[28]
	23–33		Sweden	HPLC	Cultivar and germination study	[50]
Buckwheat (*Fagopyrum esculentum*)	30		United States	–	USDA FoodData Central	[29]
	54	(flour)				
	42	(dried)				
	19		Japan	–	Standard Tables of Food Composition in Japan	[28]
	51	(flour)				
	8	(cooked)				
Pearl millet (*Pennisetum glaucum*)	24–73	(flour)	Burkina Faso	Microbio	Cultivar and processing studies	[30]
Corn (*Zea mays*)	28		Japan	–	Standard Tables of Food Composition in Japan	[28]
	19		United States	–	USDA FoodData Central	[29]
Corn	18		Brazil	–	Brazilian Food Composition Table	[44]
Rice (*Oryza sativa*)	3–12		Japan	–	Standard Tables of Food Composition in Japan	[28]
	11	(flour)	Egypt	HPLC	Regional analytical study	[30]
	4	(flour)	Brazil	–	Brazilian Food Composition Table	[44]
**Nuts and seeds**						
Peanut (*Arachis hypogaea*)	240–246		Spain	–	USDA FoodData Central	[29]
	97	(dried)	United States	–		[29]
	76	(dried)	Japan	–	Standard Tables of Food Composition in Japan	[28]
Sunflower seed (*Helianthus annuus*)	227	(dried)	United States	–	USDA FoodData Central	[29]
Hazelnut (*Corylus avellana*)	113		United States	–		[29]
	88	(dried)				
Walnut (*Juglans regia*)	98	(dried)	United States	–		[29]
Almond (*Prunus dulcis*)	44		United States	–	USDA FoodData Central	[29]
	35	(dried)				
	65	(dried)	Japan	–	Standard Tables of Food Composition in Japan	[28]
Cashew (*Anacardium occidentale)*	25		United States	–	USDA FoodData Central	[29]
	69	(dried)				
	25		Brazil	–	Brazilian Food Composition Table	[44]

^1^ Approximate values represent selected records from national databases; ranges represent variation among cultivars, samples, locations, or preparation methods. ^2^ Values are expressed as total folate in µg/100 g of edible food unless otherwise specified. Values reported for raw, flour, or dried foods should not be compared without considering moisture and processing. ^3^ Analytical methods: HPLC, high-performance liquid chromatography; Microbio, microbiological assay.

**Table 7:** Folate contents in individual foods from animal sources ^1^.

Food Source	Folate ^2^ (µg/100 g)		Geographical Origin	Method ^3^	Survey Program, Database, or Study	Ref.
**Meat, fish, and organ meats**						
Chicken liver	1300		Japan	–	Standard Tables of Food Composition in Japan	[28]
	781		Poland	–	Market-sample and cooking study	[54]
	455–780	(cooked)				
	608		Brazil	–	Brazilian Food Composition Table	[44]
	498	(fried)				
	588		United States	–	USDA FoodData Central	[29]
	578	(simmered)				
	560	(fried)				
Beef liver	1000		Japan	–	Standard Tables of Food Composition in Japan	[28]
Beef liver (*Bos taurus*)	290		United States	–	USDA FoodData Central	[29]
	260	(fried)				
	253	(braised)				
Beef liver	285		Brazil	–	Brazilian Food Composition Table	[44]
	254	(fried)				
Pork liver	810		Japan	–	Standard Tables of Food Composition in Japan	[28]
	212		United States	–	USDA FoodData Central	[29]
	163	(braised)				
Fish	3–13		Finland	LC Recipe calculation	Animal-food analytical study Fineli—Finnish Food Composition Database	[55,56]
	12	(fried)				
Other meat	3–13		Finland	LC	Animal-food analytical study	[55]
**Eggs and dairy products**						
Whole chicken egg	79–114		Poland	HPLC	Farming-system and retail-egg survey	[57]
	49		Japan	–	Standard Tables of Food Composition in Japan	[28]
	48	(boiled)				
	58	(fried)				
	47		United States	–	USDA FoodData Central	[29]
	51	(fried)				
	46		Brazil	–	Brazilian Food Composition Table	[44]
	64	(fried)				
Cow’s milk	5		United States	–	USDA FoodData Central	[29]
	5		Japan	–	Standard Tables of Food Composition in Japan	[28]

^1^ Approximate values represent selected records from national databases; ranges represent variation among cultivars, samples, locations, or preparation methods. ^2^ Values are expressed as total folate in µg/100 g of edible food unless otherwise specified. Values reported for raw, cooked, or dried foods should not be compared without considering moisture and processing. ^3^ Analytical methods: LC, liquid chromatography; HPLC, high-performance liquid chromatography.

**Table 8:** Comparison of analytical methods for folate determination.

Analytical Methods ^1^	Strengths	Limitations	Vitamer Coverage	Most Appropriate Applications	Ref.
Microbio	Low cost High sensitivity Simple equipment Detects all active forms General laboratory operation	Unequal response to vitamers Susceptible to non-folate interference Labor-intensive and time-consuming Requires microbiological expertise Low precision and poorly standardized	Total folate, cannot distinguish individual vitamers	Population folate status; food composition; low-resource labs	[1,10,61]
HPLC	Vitamer-specific quantification High sensitivity with fluorescence detection Low interference from food enzymes	High cost Low sensitivity with UV detection Requires standards for all vitamers Total folate often lower than by microbiological assay Requires extensive sample purification	Main vitamers (e.g., THF, 5-MTHF); detector dependent	Routine food folate analysis	[61,69]
LC-MS/MS	High precision High sensitivity Vitamer-specific quantification Isotope standards correct for losses	High cost Technically demanding Complex sample preparation Vitamer interconversion	Broad range of individual vitamers	Vitamer-level research	[1,10]
RPBA and EPBA	Rapid Low cost Little equipment or expertise required	Unequal response to vitamers Unsuitable for di- and polyglutamates Variation between commercial kits	Mainly folic acid; underestimates natural folates	Folic acid in fortified foods	[1]

^1^ Microbio, microbiological assay; HPLC, high-performance liquid chromatography; LC-MS/MS, liquid chromatography–tandem mass spectrometry; RPBA, radioprotein-binding assay; EPBA, enzyme protein-binding assay; THF, tetrahydrofolate; 5-MTHF, 5-methyltetrahydrofolate.

**Table 9:** Measurable determinants of food folate quality and recommended analytical approaches.

Determinant	Why Measure		How to Measure ^1^	Ref.
Total folate concentration	Defines the amount of folate a food can supply and is the basis of food composition data.		Microbiological assay, HPLC, or LC-MS/MS after enzymatic extraction and deconjugation	[64,72]
Vitamer profile	Vitamers differ in stability during processing and digestion, so foods with similar total folate can deliver different amounts.		HPLC with fluorescence/UV detection; stable-isotope dilution LC-MS/MS	[41,53,61]
Polyglutamylation degree	Polyglutamates require deconjugation before absorption, and their bioavailability may be lower than that of monoglutamates, depending on the food matrix.		LC-MS/MS of intact polyglutamates without deconjugation	[73,74]
Stability during processing	Folate losses vary with processing method; heat, oxygen, pH, and storage can cause substantial, vitamer-specific losses before consumption.		Folate analysis before and after processing; kinetic modeling of degradation and diffusion	[75,76]
Bioaccessibility	Folate must be released from the food matrix and remain intact in the gastrointestinal tract before being absorbed.		In vitro gastrointestinal digestion followed by vitamer analysis of the digesta	[53]
Absorption	Determines how much released folate is transported across the intestinal epithelium, mainly via the PCFT, whose activity can be affected by co-ingested compounds.		Stable-isotope tracing with plasma AUC	[77,78]
Biological efficacy	Absorbed folate must improve folate status and folate-dependent function to confer a nutritional benefit.		Changes in serum folate, RBC folate, and plasma homocysteine	[1]

^1^ HPLC, high-performance liquid chromatography; LC-MS/MS, liquid chromatography–tandem mass spectrometry; 5-MTHF, 5-methyltetrahydrofolate; PCFT, proton-coupled folate transporter; AUC, area under the curve; RBC, red blood cell.

## Data Availability

Only Table 2 was analyzed in this study. Data will be available on request.
